# Indocyanine green focused near-infrared fluorescence lymphography localization in treatment of chyle leak after mastectomy with targeted left axillary sentinel node biopsy for breast cancer

**DOI:** 10.1093/jscr/rjag359

**Published:** 2026-05-21

**Authors:** Bradley C Kruithoff, Dawn Cox, Mark Cripe

**Affiliations:** Department of Surgery, Ohio Health Doctors Hospital, 5100 W Broad Street, Columbus, OH 43228, United States; Department of Surgery, Ohio Health Grant Medical Center, 111 S Grant Ave, Columbus, OH 43215, United States; Department of Breast Surgery, Ohio Health Grant Medical Center, 111 S Grant Ave, Columbus, OH 43215, United States; Department of Breast Surgery, Ohio Health Grant Medical Center, 111 S Grant Ave, Columbus, OH 43215, United States

**Keywords:** indocyanine green, breast surgery, breast cancer, chyle leak, surgery

## Abstract

We present a woman in her 60s who underwent bilateral mastectomy with left axillary targeted lymph node biopsy in the setting of high grade invasive ductal carcinoma of the left breast with metastasis to a left axillary lymph node. Her postoperative course was complicated by high output milky drainage from her left axillary surgical drain, consistent with a chyle leak, after confirmatory testing demonstrated drain fluid with high triglyceride content. The patient failed non-operative management consisting of low-fat diet and compressive measures. Ultimately, the patient returned to the operating room where indocyanine green near-infrared fluorescence lymphography was used to localized damaged lymphatic channels, which were then ligated to resolve the leak. This report outlines a unique identification method of an unusual complication and serves to inform breast surgeons who may encounter this complication.

## Introduction

Breast cancer is the most common non-cutaneous skin cancer among women in United States and is the second leading cause of cancer death among women. Roughly, 1 in 8 women will undergo breast and axillary surgery in treatment of breast cancer. Chyle leak following breast cancer surgery is a rare complication and is without formal guidelines regarding treatment and management. This report outlines a unique identification of a rare complication and serves to add to the body of literature to outline both operative and non-operative management options of chyle leak after axillary surgery.

## Case report

A women in her 60s presented to the breast surgical oncology office after she underwent screaming mammography which noted a mass in the left breast. Diagnostic mammogram and ultrasound confirmed a complex cystic mass measuring 1.3 cm with three irregular, suspicious lymph nodes. Biopsy of the breast mass and one axillary lymph node confirmed a high grade invasive duct carcinoma, estrogen, and progesterone receptor negative, Her2Neu positive. The ki67 was elevated at 64%. She underwent neoadjuvant chemotherapy receiving carboplatin, taxotere, Herceptin, and perjeta every 3 weeks for six doses. After discussions regarding surgical treatment, the patient opted for a mastectomy with targeted sentinel lymph node biopsy.

At the time of surgery, the breast was removed along with the previously biopsied lymph node. Targeted lymph node excision was confirmed by interoperative imaging and final pathology confirming the biopsy site. Two additional sentinel lymph nodes were removed. There was a complete pathological response in both the breast and three axillary sentinel lymph nodes. A drain was placed along the mastectomy incision and the patient was discharged home the same day.

**Figure 1:**
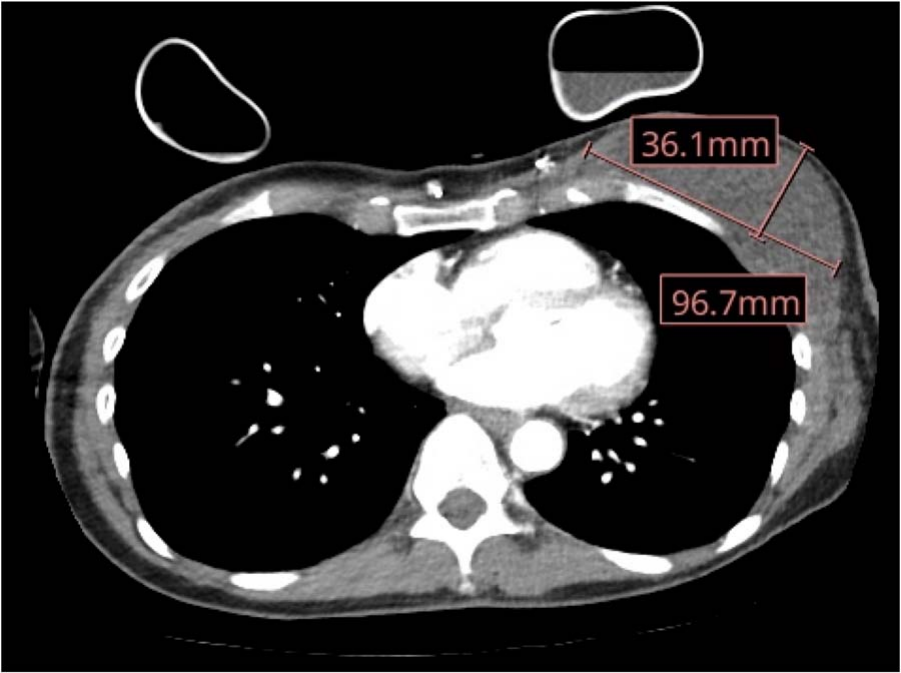
CT chest with IV contrast demonstrating a 9.6 × 3.6 cm fluid collection in the left mastectomy bed.

On postoperative day (POD) 8, the patient noticed increased left drain output with approximately 220 ml/day. On POD 10, she had no drain output with associated left chest swelling and presented to the emergency department for evaluation. Computed tomography (CT) of the chest was obtained that demonstrated a significant fluid collection in the left chest without air-fluid levels or other signs of infection (Fig. 1). Her surgical drain was stripped and a clot was removed which then resulted in significant white, milky drainage.

Upon review by the surgeon there was concern for a chyle leak, and drain fluid was sent for analysis. Results noted elevated triglyceride levels at 746 mg/dl in addition heavy growth of enterococcus fecalis and light growth of *Acinetobacter ursingii*. She was placed on a low-fat diet, limited physical activity, and antibiotics. Despite this, her drain output remained elevated with over 700 ml daily, and the patient was admitted to the hospital for hypotension secondary to the fluid losses.

**Figure 2:**
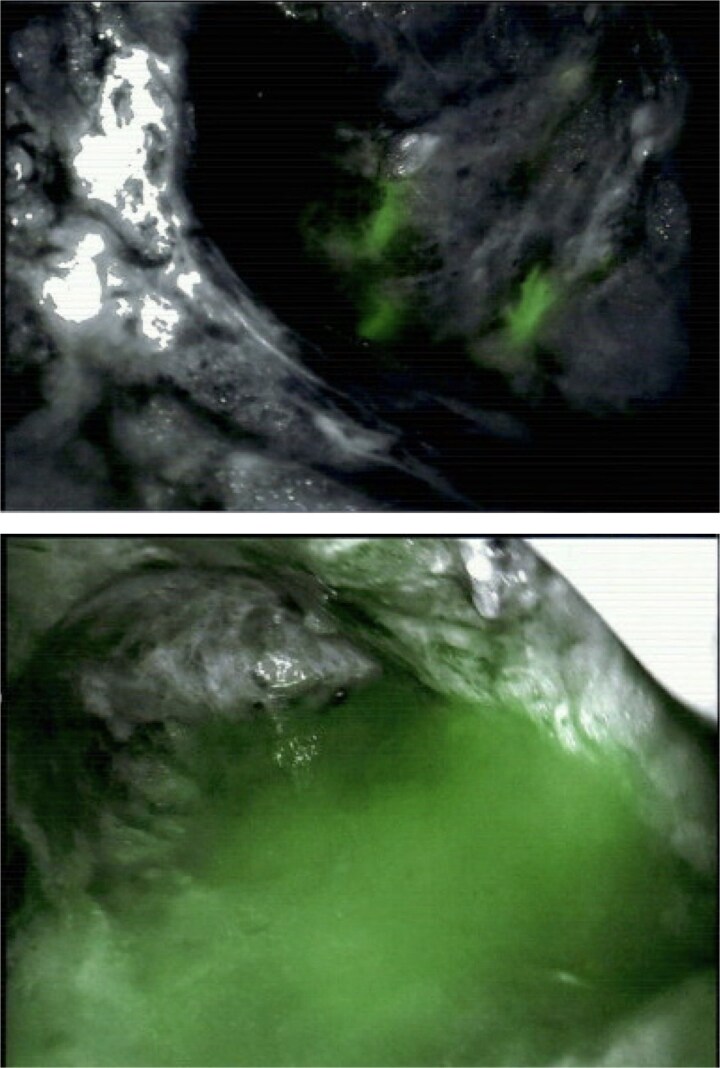
DaVinci Xi surgical camera equipped with Firefly imaging system utilizing near-infrared fluorescence lymphography to identify damaged, leaking lymphatics in the axilla.

Given failure of conservative management, the patient was brought back to the operating room for more definitive treatment. In order to localize the damaged lymphatics, 1.25 mg of indocyanine green (ICG) was injected into the bilateral inner thighs and massaged for 5 min. The prior axillary incision was opened, and a DaVinci Xi surgical camera, equipped with Firefly imaging system, was utilized to assist with near-infrared fluorescence lymphography. Damaged lymphatic channels leaking ICG were identified emanating from the lateral and medial axilla and were ligated using an energy device (Fig. 2). Additionally, a large, transected lymphatic duct was identified leaking ICG and was ligated using 5 mm surgical clips. Two new drains were placed in the axilla and the surgical site was closed. The patient was discharged from the hospital on POD 1 having tolerated the surgery well.

The patient returned to the breast surgery office POD 13 from the second surgery with significantly decreased surgical drain output with less than 10 ml output daily. One drain was removed, with the subsequent drain removed on POD 18 for continued low-output. The patient was transitioned to a regular diet and has not had reaccumulation of the fluid collection. In regards to her breast cancer, she will continue with standard postoperative surveillance and adjuvant treatment as outlined per NCCN.

## Discussion

Chyle leak is a well-described complication following head and neck, thoracic, and upper gastrointestinal surgery [1]. In respect to axillary surgery, the reported incidence is incredibly low, ranging from 0.35% to 0.86%. The majority of chyle leaks after axillary surgery occur on the left, however, a small minority develop on the right [2]. A total of 41 cases of chyle leak following axillary surgery have been previously described, mainly in patients undergoing mastectomy with axillary node dissection in the setting of breast cancer [2]. Of those with postoperative chyle leaks, the vast majorly are managed conservatively with medication and drainage procedures; however, roughly 25% of cases require surgical exploration [3]. Surgical indications for chyle leak include failure of non-operative management, high-output (typically >500 ml to 1 l/day) symptomatic fluid losses, and symptomatic nutritional deficiencies. Surgical options include direct ligation of the offending lymphatic vessels, formal ligation of the thoracic duct, fibrin glue agents, or muscle flaps to seal the area [4]. Additionally, minimally invasive methods such as embolization or ablation of the thoracic duct have been described.

This case outlines a rare postoperative complication of thoracic duct and lymphatic leak after targeted axillary lymph node dissection in the setting of locally invasive left breast cancer. Using near-infrared fluorescence lymphography with indocyanine green, we describe a methodical and systematic procedure to identify and treat the damaged lymphatics contributing to a chyle leak. This is a highly effective and innovative method to treat this surgical complication and has yet to be described in the context of oncologic breast surgery. This is a safe and durable technique that can be used for similar patients presenting with postoperative chyle leak after axillary surgery.
